# Cardiovascular dynamics of Canadian Indigenous peoples

**DOI:** 10.1080/22423982.2017.1421351

**Published:** 2018-02-06

**Authors:** Heather J. A. Foulds, Shannon S. D. Bredin, Darren E. R. Warburton

**Affiliations:** ^a^ Cardiovascular Physiology and Rehabilitation Laboratory, University of British Columbia, Vancouver, Canada; ^b^ Experimental Medicine Program, Faculty of Medicine, University of British Columbia, Vancouver, BC, Canada; ^c^ Physical Activity Promotion and Chronic Disease Prevention Unit, Vancouver, BC, Canada

**Keywords:** American Indian, First Nation, Doppler echocardiography, cardiac, echocardiography, left ventricular function, left ventricular mass, left ventricular systolic function

## Abstract

Limited understanding of Indigenous adults’ cardiovascular structure and function exists despite high rates of cardiovascular disease. This investigation characterised cardiovascular structure and function among young Indigenous adults and compared to age- and sex-matched European descendants. Echocardiographic assessments included apical two- and four-chamber images, parasternal short-axis images and Doppler. Analyses included cardiac volumes, dimensions, velocities and strains. Cardiovascular structure and function were similar between Indigenous (n=10, 25 ± 3 years, 4 women) and European-descendant (n=10, 24 ± 4 years, 4 women,) adults, though European descendants demonstrated greater systemic vascular resistance (18.19 ± 3.94 mmHg∙min^−1^∙L^−1^ vs. 15.36 ± 2.97 mmHg∙min^−1^∙L^−1^, p=0.03). Among Indigenous adults, women demonstrated greater arterial elastance (0.80 ± 0.15 mmHg·mL^−1^·m^−2^ vs. 0.55 ± 0.17 mmHg·mL^−1^·m^−2^, p=0.02) and possibly greater systemic vascular resistance (17.51 ± 2.20 mmHg∙min^−1^∙L^−1^ vs. 13.93 ± 2.61 mmHg∙min^−1^∙L^−1^, p=0.07). Indigenous men had greater cardiac size, dimensions and output, though body size differences accounted for cardiac size differences. Similar cardiac rotation and strains were observed across sexes. Arterial elastance and cardiac size were different between Indigenous men and women while cardiovascular structure and function may be similar between Indigenous and European descendants.

## Introduction

Cardiovascular disease is a significant health concern, especially among Indigenous populations [1]. In Canada, cardiovascular disease affects 7.1% of Indigenous adults and 5.0% of the general Canadian population [2]. Indigenous adults in Canada continue to face rapidly growing burdens of cardiovascular disease morbidity and mortality [3]. Cardiovascular disease development includes changes in cardiovascular structure and function, reduced cardiac perfusion and subendocardial coronary blood supply, increased afterload and cardiac hypertrophy [4,5]. These changes occur prior to clinical cardiovascular disease [6].

Cardiovascular structure and function have been investigated among older American Indians [7]. Previous cardiovascular assessments among Indigenous populations evaluated middle-aged and older individuals, with particular attention on those with chronic health conditions [7–9]. Further, assessments of cardiac strain and elastance among Indigenous populations, and direct comparisons of cardiovascular function between Indigenous and non-Indigenous populations have not been conducted. Among US Indigenous adults, left ventricular (LV) mass was related to demographic and haemodynamic variables [7], and hypertrophy with genotype differences in renin–angiotensin genes [9].

Previous investigations of Indigenous populations in Canada from the same province have identified sex differences among Indigenous adults which were not identified among other ethnic groups, including diabetes, physical inactivity and the prevalence of cardiovascular disease [10]. Conversely, a previous investigation identified sex differences in rates of high total cholesterol and low high-density lipoprotein cholesterol among other ethnic groups, which were not identified among Indigenous adults [10]. Ethnic differences in cardiovascular structure and function have been identified among African-American and Asian-American populations, and these differences may contribute to varying rates of cardiovascular disease among ethnic groups [11–13]. However, assessments of ethnic differences in cardiovascular structure and function between Indigenous and non-Indigenous adults have not been conducted.

Assessments of cardiovascular structure and function have not been investigated among Indigenous populations in Canada or younger Indigenous adults. The objective of this investigation was to characterise cardiac measures including LV dimensions and volumes, systolic and diastolic function, arterial–ventricular coupling and strains among young Indigenous adults, and compare to age- and sex-matched European-descendant adults. Greater cardiac size and volumes were hypothesised among Indigenous men compared to women, and Indigenous compared to European-descendant adults.

## Methods

### Participants and ethical approval

Ethics approval was obtained through the Clinical Research Ethics Board at the University of British Columbia in accordance with the ethical standards of the institution and with the 1964 Helsinki Declaration and its later amendments or comparable ethical standards, and written informed consent was obtained prior to data collection. Additionally, Indigenous elders from the community reviewed and approved the project prior to commencing. Indigenous and European-descendant adults were recruited from the University of British Columbia campus. Indigenous participants were recruited through existing relationships within the university. Included individuals were healthy, 19–40 year olds who have not been diagnosed with cardiovascular disease or diabetes, do not take medications for cardiovascular disease or diabetes, are able to participate in physical activity and are not pregnant. Testing was conducted with the assistance of Indigenous students.

### Study setting and population

From February to August 2013, Indigenous and European-descendant adults, 21–33 years, underwent cardiovascular assessments. All assessments were conducted at the University of British Columbia in the Cardiovascular Physiology and Rehabilitation laboratory. Participants self-declared their ethnicity and ancestral nations through a self-survey questionnaire. Participants were asked to describe their family’s ethnicity as well as mother and father’s ethnicities. Indigenous participants self-declared either First Nations or Métis ancestry, with no minimum blood quantum or status requirements. Individuals reporting First Nations or Métis ethnicity, with or without additional ethnicity mixtures, were considered Indigenous participants. European participants self-declared ethnicity only as European and did not identify any other mixed race ancestry.

### Individual characteristic and blood pressure

Individual characteristics collected included age, sex, education level, income, employment, self-reported health measures including diabetes and cardiovascular disease and smoking. Anthropometric measures of height, body mass and waist circumference were assessed according to standardised protocols [14]. World Health Organization cut-off points were used for obesity classifications [15], using European obesity definitions as these definitions are correlated with body fat among Indigenous populations [16]. Following 5 min of supine rest, supine resting blood pressure was measured three times on the left arm, with 1 min rest between each measurement, using an automated blood pressure monitor (HDI/Pulsewave CR-2000 Cardiovascular Profiling System, Egan, MN, USA). Hypertension was defined as the use of antihypertension medication or average measurements of ≥140 mmHg systolic or ≥90 mmHg diastolic [17].

### Echocardiography and data analysis

A trained clinical echocardiography sonographer using a portable ultrasound (*Vivid I*, GE Healthcare, Wauwatosa, WI, USA), with simultaneous limb-lead electrocardiography and a 2.5-MHz phased-array transducer, conducted the cardiovascular assessments with an intra-operator coefficient of variation of 3.8%. Participants were imaged in the left lateral decubitus position to obtain M-mode, two-dimensional and Doppler echocardiography images. Conventional apical two- and four-chamber views were obtained to quantify LV volumes, longitudinal strain and strain rate [18]. Parasternal short-axis images at the papillary muscle level were obtained to determine rotation, rotation rate, radial and circumferential strain and strain rates. M-mode images were used to determine LV posterior wall thickness, internal diameter, septal wall thickness and LV mass. Doppler recordings included pulsed Doppler with the cursor at mitral leaflets tips to quantify early (E) and late (A) ventricular filling velocities, and tissue Doppler imaging of septal and lateral mitral annular tissue velocity (Eʹ). Each image recorded at least three consecutive cardiac cycles at high strain rates (80–90 frames per second).

Offline analysis was used to determine ventricular volumes, dimensions, tissue velocities and strains (EchoPAC, GE Healthcare, v. 110.1.1). All measurements and calculations averaged three consecutive cardiac cycles. Body surface area (BSA) was calculated from height and weight [19]. Linear and Simpson’s biplane methods were used to determine LV mass and volumes, respectively [18]. LV mass was also indexed for height^2.7^. Stroke volume index (SVI) and cardiac output index were determined by indexing stroke volume and cardiac output for BSA. Ejection fraction was determined from stroke volume as a percentage of end diastolic volume. Ventricular diameters were used to determine fractional shortening, expressed as a percentage. End systolic wall stress, a measure of afterload, was calculated from 0.334 × systolic blood pressure × LV end systolic diameter/(LV systolic posterior wall thickness × (1 + LV systolic posterior wall thickness/LV end systolic diameter)) [20]. Relative wall thickness was determined as twice the diastolic posterior wall thickness/internal diameter [8]. Diastolic function was evaluated using E/A ratio to determine the proportion of filling occurring early in the cardiac cycle.

End systolic pressure was determined as 0.9 × brachial systolic blood pressure [21]. Arterial elastance was calculated from end systolic pressure/stroke volume. This method of measuring arterial elastance is found to provide good agreement with measurements from pressure–volume data and arterial impedance [21,22]. Ventricular elastance was calculated as end systolic pressure/end systolic volume. Both arterial and ventricular elastance were indexed for BSA to calculate E_A_I and E_LV_I, respectively. Arterial–ventricular coupling was calculated as E_A_I/E_LV_I. Arterial stiffness, and its inverse, arterial compliance were calculated from the ratios of pulse pressure to stroke volume and stroke volume to pulse pressure, as measures of systolic function [23]. Arterial compliance and arterial stiffness can be reliably evaluated through echocardiography [23–26].

Speckle-tracking analysis was applied to quantify strain and strain rates from four-chamber (longitudinal) and parasternal short-axis (radial and circumferential) images. This analysis was conducted on the entire width of the myocardium and separately on the endocardial (innermost) and epicardial (outermost) layers of myocardium.

### Sample size calculations

Power calculations indicated sex differences in measures such as wall stress, LV mass and ejection fraction/end diastolic volume can be determined with sample sizes of 4–12 per sex among young normally active adults, with 4 participants per sex required for wall stress, 9 participants per sex for LV mass/BSA, and 12 participants required for ejection fraction/end diastolic volume [27]. Comparisons between ethnicities may require larger sample sizes, with samples of less than 10 participants needed for LV mass/BSA, septal wall thickness and LV posterior wall thickness among one study [28]. A priori sample size calculations based on another investigation suggest that ethnic differences may be identified with as few as 8 participants per ethnic group for LV mass, and as few as 22–23 participants per ethnic group for stroke volume and LV mass/BSA [12].

### Statistical analysis

Statistical analyses were performed using Statistica 9.0 (Stats Soft, Tulsa, OK, USA). Continuous variables were reported as mean and standard deviation, while categorical and binary factors were reported as percentages and counts. Pearson χ^2^ test were used to compare categorical variables between sexes and ethnic groups. Differences between and within ethnic groups were conducted using two-way factorial analysis of variance with Tukey’s HSD for unequal sample size post-hoc test. Significance was set at p<0.05 for all analyses.

## Results

Ten Indigenous adults, from a recruited 16, completed the assessment. Individuals not completing the assessment were unable to attend or complete the assessment due to personal/family challenges. Ten age- and sex-matched European-descendant adults formed a comparison group. Matching was stratified by sex and within 1 year. Similar demographics were identified between Indigenous men and women, and Indigenous and European-descendant participants (Table 1). Participants were young adults, generally single, well-educated and employed, with incomes below $20,000 per year. Indigenous men were taller and heavier with greater waist circumferences than Indigenous women, though with similar obesity rates. Similar sex differences were observed among European-*descendant adults, though waist circumference was not different between European-descendant men and women. Indigenous and European-descendant adults were of similar height, body mass and BSA, with similar obesity rates, though Indigenous adults were found to have greater waist circumferences. Blood pressures were similar across both ethnic groups and Indigenous sexes, and generally fell within normal ranges. However, heart rates were greater among Indigenous adults. All participants reported being free of diabetes and cardiovascular disease.

**Table 1.** Demographic characteristics of participants, by sex mean ± SD, *n* (%).

	Indigenous men (*n*=6)	Indigenous women (*n*=4)	*p*-Value	Indigenous (*n*=10)	European men (*n*=6)	European women (*n*=4)	European descent (*n*=10)	*p*-Value^a^	*p*-Value^b^
Age (years)	26 ± 3	23 ± 3	0.10	25 ± 3	25 ± 5	22 ± 2	24 ± 4	0.17	0.49
Female, *n* (%)	–	–	–	4 (40.0)	–	–	4 (40.0)	–	1.00
First Nations, *n* (%)	2 (33.3)	3 (75.0)	0.24	5 (50.0)	–	–	–	–	–
Métis, *n* (%)	4 (66.7)	1 (25.0)	0.24	5 (50.0)	–	–	–	–	–
Single, *n* (%)	5 (83.3)	2 (50.0)	0.31	7 (70.0)	5 (83.3)	3 (75.0)	8 (80.0)	0.79	0.63
More than high school education, *n* (%)	3 (50.0)	4 (100.0)	0.31	7 (70.0)	6 (100.0)	4 (100.0)	10 (100.0)	1.00	0.06
Employed, *n* (%)	5 (83.3)	4 (100.0)	0.45	9 (90.0)	4 (66.7)	4 (100.0)	8 (80.0)	0.24	0.56
Annual income <$20,000 per year, *n* (%)	4 (66.7)	4 (100.0)	0.24	8 (80.0)	3 (50.0)	2 (50.0)	5 (50.0)	1.00	0.18
Smoker, *n* (%)	2 (33.3)	0 (0.0)	0.24	2 (20.0)	0 (0.0)	0 (0.0)	0 (0.0)	1.00	0.15
Hypertension, *n* (%)	0 (0.0)	0 (0.0)	1.00	0 (0.0)	1 (16.7)	0 (0.0)	1 (10.0)	0.36	0.33
Height (cm)	181.1 ± 6.9	166.5 ± 5.4	0.04	175.2 ± 9.6	179.6 ± 5.6	165.2 ± 4.7	173.8 ± 9.0	0.03	0.78
Body mass (kg)	88.3 ± 11.4	62.9 ± 6.2	0.01	78.1 ± 16.0	80.2 ± 7.4	63.0 ± 5.6	73.3 ± 2.1	0.02	0.29
BSA (m^2^)	2.1 ± 0.2	1.7 ± 0.1	0.01	1.9 ± 0.2	2.0 ± 0.1	1.7 ± 0.1	1.9 ± 1.7	0.01	0.40
Waist circumference (cm)	93.8 ± 7.3	73.9 ± 5.1	0.003	85.8 ± 12.0	80.5 ± 5.3	76.1 ± 5.0	78.7 ± 4.9	0.22	0.02
Overweight, *n* (%)	3 (50.0)	1 (25.0)	0.49	4 (40.0)	3 (50.0)	1 (25.0)	4 (40.0)	0.49	1.00
Obesity, n (%)	1 (16.7)	0 (0.0)	0.45	1 (10.0)	0 (0.0)	0 (0.0)	0 (0.0)	1.00	0.33
Systolic blood pressure (mmHg)	118.2 ± 13.3	112.0 ± 5.4	0.40	115.7 ± 10.9	124.7 ± 10.9	112.5 ± 7.9	119.8 ± 11.2	0.10	0.42
Diastolic blood pressure (mmHg)	63.3 ± 12.0	59.3 ± 5.5	0.56	61.7 ± 9.7	71.7 ± 14.7	63.0 ± 5.2	68.2 ± 12.2	0.29	0.20
Heart rate (beats·min^−1^)	62.4 ± 9.9	59.5 ± 11.6	0.56	61.2 ± 10.1	52.8 ± 3.1	55.5 ± 4.9	53.9 ± 3.9	0.28	0.045

^a^Sex differences among European descendants. ^b^Ethnic comparison. BSA: body surface area; SD: standard deviation.

**Figure 1.**
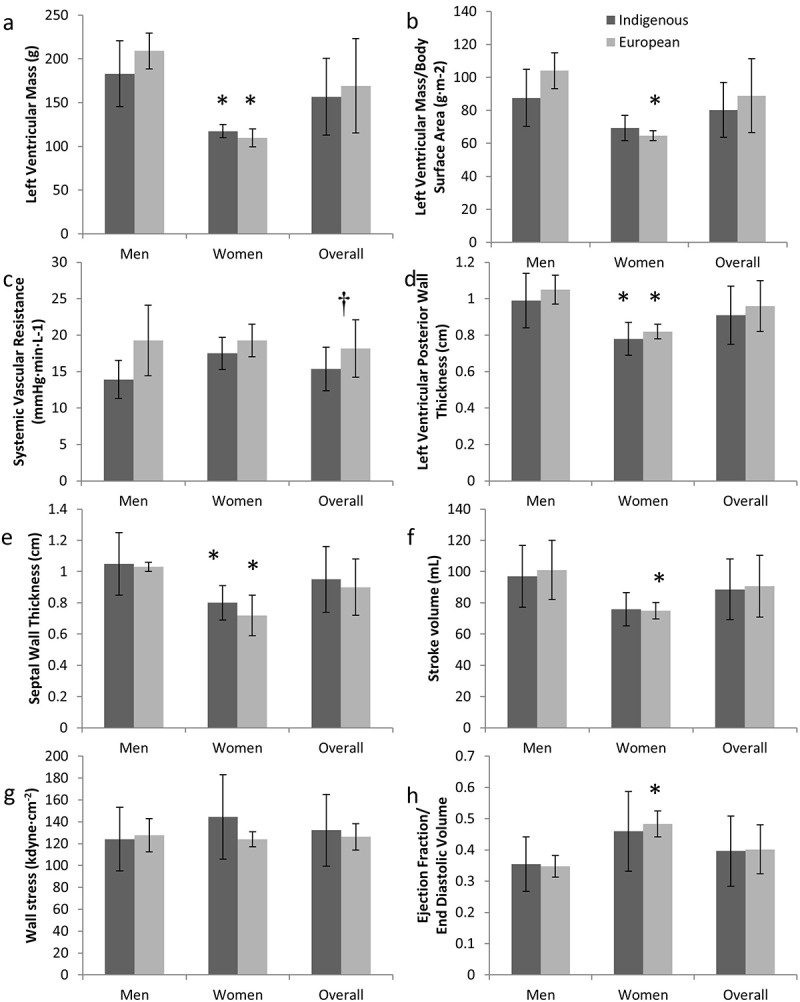
Left ventricular mass (a), left ventricular mass indexed to body surface area (b), systemic vascular resistance (c), left ventricular posterior wall thickness (d), septal wall thickness (e), stroke volume (f), wall stress (g) and ejection fraction:end diastolic volume rate (h) among Indigenous and European adults. *Indicates significant difference from men of same ethnicity; †indicates ethnic difference.

Resting ECG measurements among both ethnicities represented typically expected ECG results; ECG abnormalities suggesting underlying cardiovascular disease were not observed. Sex differences in LV mass were identified within Indigenous adults, though these differences were eliminated when adjusting for body size (Figure 1(a,b) and Table 2). Sex differences between European-descendant men and women were observed for most cardiac size and elastance measurements, though no differences were observed for systemic vascular resistance (Figure 1(c –e) and Table 2). Similarities in LV mass were found between ethnic groups. Dimensions of LV relative wall thicknesses and systolic and diastolic volumes were similar between Indigenous men and women, and across ethnic groups. However, larger posterior and septal wall thicknesses were identified among Indigenous men compared to women. Posterior and septal wall thicknesses were similar between ethnic groups (Figure 1(d,e)). Arterial–ventricular coupling was similar across ethnic groups and between sexes within ethnic groups. However, Indigenous women were found to have greater arterial elastance than Indigenous men. Ventricular elastance may not be different between Indigenous men and women (E_A_I: p=0.02; E_LV_I: p=0.09). Systemic vascular resistance was found to be greater among European descendants (p=0.03) than Indigenous adults, and a trend was identified within Indigenous adults with women possibly having greater resistance compared to Indigenous men (p=0.07).

**Table 2.** Resting cardiac measurements and elastance of participants, by sex mean ± SD.

	Indigenous men (*n*=6)	Indigenous women (*n*=4)	*p*-Value	Indigenous (*n*=10)	European men (*n*=6)	European women (*n*=4)	European descent (*n*=10)	*p*-Value^a^	*p*- value^b^
LV mass/height^2.7^ (g·m^−2.7^)	36.9 ± 7.8	29.8 ± 3.5	0.13	34.0 ± 7.1	43.3 ± 6.1	28.3 ± 2.2	37.3 ± 9.0	0.04	0.35
E_A_I (mmHg·mL^−1^·m^−2^)	0.55 ± 0.17	0.80 ± 0.15	0.02	0.65 ± 0.20	0.57 ± 0.13	0.80 ± 0.06	0.66 ± 0.15	0.01	0.34
E_LV_I (mmHg·mL^−1^·m^−2^)	0.75 ± 0.24	1.14 ± 0.40	0.08	0.91 ± 0.34	0.81 ± 0.12	1.21 ± 0.18	0.97 ± 0.25	0.02	0.31
E_A_I/E_LV_I	0.74 ± 0.04	0.73 ± 0.12	0.75	0.74 ± 0.08	0.71 ± 0.09	0.67 ± 0.07	0.69 ± 0.08	0.41	0.22
*Systolic*									
LVID (cm)	3.30 ± 0.40	3.06 ± 0.38	0.37	3.20 ± 0.39	3.50 ± 0.29	2.92 ± 0.25	3.27 ± 0.40	0.02	0.72
LVID/BSA (m·m^−2^)	15.8 ± 1.6	18.1 ± 3.2	0.17	16.7 ± 2.5	17.6 ± 2.1	17.2 ± 0.8	17.5 ± 1.6	0.71	0.45
End systolic volume (mL)	72.3 ± 16.8	55.4 ± 13.4	0.13	65.6 ± 17.1	70.1 ± 5.9	49.8 ± 4.9	62.0 ± 11.7	0.01	0.59
End systolic volume/BSA (mL·m^−2^)	34.6 ± 7.4	32.3 ± 6.4	0.63	33.7 ± 6.7	35.2 ± 3.3	29.4 ± 2.2	32.9 ± 4.1	0.02	0.76
*Diastolic*									
LVID (cm)	4.86 ± 0.34	4.56 ± 0.19	0.15	4.74 ± 0.32	5.19 ± 0.14	4.49 ± 0.45	4.91 ± 0.46	0.08	0.33
Relative wall thickness	0.41 ± 0.08	0.34 ± 0.05	0.16	0.38 ± 0.07	0.40 ± 0.02	0.37 ± 0.05	0.39 ± 0.04	0.29	0.83
End diastolic volume (mL)	169.3 ± 33.5	131.3 ± 22.8	0.08	154.1 ± 36.0	171.1 ± 24.2	124.7 ± 8.5	152.5 ± 30.4	0.04	0.52
End diastolic volume/BSA (mL·m^−2^)	81.1 ± 16.2	76.7 ± 9.8	0.65	79.3 ± 13.5	86.1 ± 13.5	73.7 ± 3.2	81.1 ± 12.1	0.08	0.76

^a^Sex differences among European descendants; ^b^Ethnic comparison. BSA: body surface area; E_A_I: arterial elastance indexed for body surface area; E_LV_I: ventricular elastance indexed for BSA; LV: left ventricular; LVID: left ventricular internal diameter; SD: standard deviation.

Cardiac output was greater among Indigenous men than women, though no difference was identified once adjustments for body size were incorporated (Table 3). Stroke volume among Indigenous participants may not have been different between sexes (Figure 1(f); p=0.09). Sex differences among European descendants were similar to that of Indigenous adults, with significant differences in stroke volume (Figure 1(f)). Cardiac output and stroke volume were similar among across ethnic groups (Figure 1(f) and Table 3). Systolic functional measurements were similar across Indigenous sexes and ethnic groups, including ejection fraction, wall stress, ejection fraction:end diastolic volume ratios, velocities, arterial compliance, arterial stiffness and fractional shortening (Figure 1(g,h) and Table 3). Diastolic function and velocities were also similar across ethnic groups and Indigenous sexes (Table 3). Table 4 identifies similar strain, strain rates, rotation and rotational velocities between ethnic groups and sexes.

**Table 3.** Cardiac function of participants, by sex mean ± SD.

	Indigenous men (*n*=6)	Indigenous women (*n*=4)	*p*-Value	Indigenous (*n*=10)	European men (*n*=6)	European women (*n*=4)	European descent (*n*=10)	*p*-Value^a^	*p*-Value^b^
*Systolic*									
SVI (mL·m^−2^)	46.5 ± 9.0	44.4 ± 4.5	0.69	45.7 ± 7.2	50.9 ± 10.4	44.2 ± 2.5	48.2 ± 8.6	0.22	0.48
Cardiac output (L·min^−1^)	5.9 ± 0.7	4.4 ± 0.6	0.01	5.3 ± 1.0	5.3 ± 1.1	4.2 ± 0.6	4.9 ± 1.1	0.07	0.20
Cardiac output index (L·min^−1^·m^−2^)	2.8 ± 0.3	2.6 ± 0.4	0.35	2.7 ± 0.3	2.7 ± 0.6	2.5 ± 0.3	2.6 ± 0.5	0.42	0.42
Ejection fraction (mL)	57.4 ± 1.3	58.2 ± 4.2	0.67	57.7 ± 2.7	58.7 ± 3.0	60.1 ± 2.4	59.3 ± 2.7	0.41	0.22
Fractional shortening (%)	16.6 ± 4.8	21.0 ± 4.5	0.19	18.3 ± 5.0	16.8 ± 3.4	21.2 ± 2.3	18.5 ± 3.7	0.05	0.92
Pulse pressure/SVI (mmHg· m^2^·mL^−1^)	1.23 ± 0.31	1.19 ± 0.04	0.79	1.21 ± 0.23	1.08 ± 0.36	1.12 ± 0.07	1.09 ± 0.27	0.79	0.31
SVI/pulse pressure (mL·mmHg^−1^·m^−2^)	0.85 ± 0.17	0.84 ± 0.03	0.97	0.85 ± 0.13	1.10 ± 0.63	0.90 ± 0.06	1.02 ± 0.48	0.47	0.29
*Diastolic*									
E (m·s^−1^)	0.73 ± 0.13	0.79 ± 0.10	0.49	0.75 ± 0.12	0.68 ± 0.18	0.80 ± 0.11	0.72 ± 0.16	0.23	0.68
A (m·s^−1^)	0.33 ± 0.08	0.30 ± 0.08	0.64	0.32 ± 0.08	0.27 ± 0.07	0.32 ± 0.05	0.29 ± 0.07	0.20	0.35
Eʹ septal (m·s^−1^)	0.23 ± 0.03	0.22 ± 0.02	0.80	0.22 ± 0.03	0.21 ± 0.03	0.23 ± 0.03	0.22 ± 0.03	0.29	0.68
Eʹ lateral (m·s^−1^)	0.24 ± 0.04	0.24 ± 0.00	0.91	0.24 ± 0.03	0.25 ± 0.04	0.26 ± 0.02	0.25 ± 0.03	0.68	0.34
E/A	2.38 ± 0.72	2.71 ± 0.40	0.44	2.51 ± 0.61	2.75 ± 0.98	2.58 ± 0.37	2.68 ± 0.77	0.73	0.58
E/Eʹ septal	3.26 ± 0.55	3.57 ± 0.29	0.80	3.38 ± 0.47	3.21 ± 0.51	3.52 ± 0.75	3.33 ± 0.60	0.87	0.84
E/Eʹ lateral	3.07 ± 0.38	3.32 ± 0.41	0.36	3.17 ± 0.39	2.72 ± 0.54	3.13 ± 0.59	2.88 ± 0.57	0.54	0.20

^a^Sex differences among European descendants; ^b^Ethnic comparison. A: late ventricular diastolic filling velocity; E: early ventricular diastolic filling velocity; Eʹ: mitral annular tissue velocity; SD: standard deviation; SVI: stroke volume indexed for body surface area.

**Table 4.** Cardiac mechanics of participants, by sex mean ± SD.

	Indigenous men (*n*=6)	Indigenous women (*n*=4)	*p*-Value	Indigenous (*n*=10)	European men (*n*=6)	European women (*n*=4)	European descent (*n*=10)	*p*-Value^a^	*p*-Value^b^
*Peak (systole)*									
Rotation (°)	4.36 ± 2.75	2.70 ± 2.50	0.36	3.70 ± 2.65	4.14 ± 1.59	2.01 ± 1.14	3.28 ± 1.74	0.05	0.69
Rotation velocity (°·s^−1^)	41.84 ± 23.43	48.34 ± 20.96	0.67	44.43 ± 21.51	47.11 ± 19.14	45.54 ± 13.96	46.48 ± 16.40	0.86	0.81
Longitudinal strain (%)	−18.37 ± 1.53	−18.82 ± 1.89	0.69	−18.55 ± 1.60	−17.66 ± 2.27	−13.95 ± 9.36	−16.17 ± 5.98	0.49	0.24
Longitudinal strain rate (s^−1^)	−1.02 ± 0.20	−0.99 ± 0.11	0.80	−1.01 ± 0.16	−0.99 ± 0.13	−0.74 ± 0.50	−0.89 ± 0.33	0.42	0.33
Radial strain (%)	24.98 ± 10.16	24.24 ± 13.17	0.92	24.68 ± 10.74	28.82 ± 13.69	28.24 ± 11.00	28.59 ± 12.02	0.94	0.45
Radial strain rate (s^−1^)	1.37 ± 0.35	1.43 ± 0.35	0.80	1.39 ± 0.30	1.21 ± 0.29	1.54 ± 0.26	1.34 ± 0.32	0.12	0.72
Circumferential strain (%)	−12.61 ± 3.55	−13.78 ± 2.97	0.60	−13.08 ± 3.21	−12.78 ± 1.91	−12.48 ± 1.90	−12.66 ± 1.81	0.83	0.73
Circumferential strain rate (s^−1^)	−0.82 ± 0.22	−0.86 ± 0.22	0.79	−0.84 ± 0.21	−0.80 ± 0.22	−0.76 ± 0.06	−0.78 ± 0.17	0.67	0.53
*Peak (diastole)*									
Rotation velocity (°·s^−1^)	−32.98 ± 12.70	−42.62 ± 15.87	0.32	−36.83 ± 14.08	−42.94 ± 14.88	−51.10 ± 10.95	−46.20 ± 13.44	0.31	0.15
Longitudinal strain rate (s^−1^)	1.40 ± 0.14	1.40 ± 0.22	0.99	1.40 ± 0.16	1.37 ± 0.37	1.19 ± 0.82	1.30 ± 0.56	0.70	0.60
Radial strain rate (s^−1^)	−1.35 ± 0.48	−1.51 ± 0.79	0.69	−1.42 ± 0.58	−1.39 ± 0.20	−1.57 ± 0.65	−1.46 ± 0.41	0.58	0.85
Circumferential strain rate (s^−1^)	0.78 ± 0.36	1.02 ± 0.29	0.29	0.88 ± 0.34	0.99 ± 0.31	0.86 ± 0.23	0.94 ± 0.27	0.46	0.66

^a^Sex differences among European descendants; ^b^Ethnic comparison. SD: standard deviation.

## Discussion

This investigation evaluated resting cardiovascular structure and function among Indigenous young adults and compared to age- and sex-matched European descendants. The present study identified sex differences in arterial elastance, while differences in LV mass and cardiac output among Indigenous adults may be accounted for by differences in body size. These findings suggest body size, and potentially obesity, may mediate sex differences in cardiovascular structure and function among Indigenous adults.

Present participants were younger than general Indigenous populations in Canada (mean 25 years vs. median 28 years), with more Métis (50%) than the Canadian average (32.3%) [29]. Indigenous participants in the current investigation reported greater education and employment, and lower chronic health conditions compared to reports among the general Indigenous populations in Canada [10,30]. Younger, healthier participants in this investigation likely experience less LV hypertrophy and heart failure than general Indigenous populations. However, as cardiovascular disease risk factors develop prior to overt cardiovascular disease, indications of LV hypertrophy may appear at a young age [4,6].

In comparison to previous investigations of middle-aged Indigenous adults free of chronic health conditions, the present adults demonstrate similar cardiovascular structure and function [8,31,32]. However, likely due to younger ages, the current sample demonstrated greater cardiac dimensions, volumes, velocities and more favourable wall stress, arterial stiffness and systemic vascular resistance than many previously evaluated Indigenous adults [8,31,32]. Sex differences identified among Indigenous adults are consistent with previous findings among Indigenous and/or young, healthy adults, with differences in LV mass, arterial elastance, arterial impedance and stroke volume [27,32,33], and differences in LV mass and cardiac output are accounted for by differences in BSA [27]. More sex differences were identified among the European-descent adults, including LV mass indexed for body size, ventricular elastance, systolic LV internal diameter, end systolic and diastolic volumes, stroke volume, fractional shortening and rotation, consistent with normal values [34,35]. Previously identified sex differences in ejection fraction [8,31], relative wall thicknesses, peak E and A velocities, and E/A ratio [32] may not have been identified in the current investigation, among either ethnic group, likely due to a small sample size. The sex differences identified among European descent but not Indigenous participants suggest differences between sexes among Indigenous populations may be of less magnitude, though larger sample sizes are likely required to evaluate sex-specific ethnic differences.

As obesity may be a mediating factor in LV hypertrophy, cardiovascular disease development and ethnic differences, evaluating cardiac structure and function indexed for BSA may be important [32]. Among published literature, only LV mass has been evaluated relative to BSA among Indigenous adults, while LV mass, systolic LV internal diameter, end systolic and diastolic volumes, and cardiac output are reported relative to BSA among non-Indigenous adults [27,34,35]. In comparison to Indigenous populations previously evaluated, LV mass was smaller among women participants in the current investigation, even after indexing for BSA and comparing to non-obese women [32]. Similarly, both Indigenous and European-descent LV mass indexed to BSA were lower than normally active adults, though European-descent men averaged within 3 g·m^−2^ [27]. Cardiac output indexed to BSA was higher among Indigenous and European-descent adults in the present study compared to normally active adults [27]. When indexed to surface area, the present study measures of systolic LV internal diameter are similar to published normative data, though sex differences were not identified among either ethnicity in this investigation [34]. Compared to published normal values, end systolic and diastolic values indexed for BSA among the present investigation were greater and sex differences were only identified among European-descent adults in the present investigation [35]. The similarity among Indigenous and European-descendant averages in the present investigation in comparison to other published samples, even after adjusting for BSA, suggests that these differences may be the result of age, regional and/or sociodemographic differences, rather than ethnic differences.

Comparisons of cardiovascular structure and function between Indigenous non-Indigenous populations have not previously been conducted. This investigation identified similar cardiovascular structure and function among Indigenous and European-descendant adults of similar age, education, income, body size and fitness level. Indigenous populations currently experience greater cardiovascular disease than other Canadian populations [10]. Similarities in resting cardiovascular structure and function between Indigenous and European-descendant young adults suggest that subclinical cardiovascular disease development may occur similarly across ethnicities. These similarities in cardiovascular structure and function may seem counterintuitive given the greater experiences of obesity, diabetes and cardiovascular disease among Indigenous populations [2,36]; however, similar or more favourable vascular structure and function have been previously identified among Indigenous populations compared to European descent [37–39]. Similarities in cardiovascular structure and function, combined with previous similarities in vascular structure and function [37–39], suggest that the differences in cardiovascular disease are rooted outside of cardiovascular physiology and genetics, as previously suggested [40,41]. Alternatively, while ethnic differences in resting cardiovascular dynamics were not identified, ethnic differences may be more apparent when evaluating ECG during maximal or near-maximal exercise stress tests which are prognostic for identifying cardiac arrhythmias and abnormalities among asymptomatic individuals with underlying cardiovascular disease [42].

### Strengths and limitations

As cardiovascular disease progression begins prior to overt cardiovascular disease [4,6], evaluating young, apparently healthy individuals is important for understanding the disease progression and risk development among this high-risk population. This investigation is the first to directly compare cardiovascular structure and function between Indigenous and non-Indigenous populations. Understanding ethnic differences and similarities is important for understanding cardiovascular disease experiences among Indigenous populations. Longitudinal strain is a correlate of LV contractility and indicates regional myocardial dysfunction and longitudinal and circumferential strains are prognostic of cardiovascular events and survival [43–45]. Further, elastance and arterial–ventricular coupling provide insight into the interaction between the arterial system and the LV, evaluating stroke work and energetic efficiency and providing indications of atherosclerotic heart disease [46]. The inclusion of these measures adds to current understanding of cardiovascular function among Indigenous populations.

This cross-sectional investigation represents a pilot investigation of cardiovascular measures among Indigenous peoples and is not able to evaluate long-term cardiovascular outcomes. As such, determinations of normative cardiovascular measures for Indigenous populations applicable for predicting long-term outcomes cannot be determined. Further research including long-term follow-up is required to determine if normative LV parameters are appropriate for this population. Larger sample sizes are required to evaluate sex-specific ethnic differences. This young, healthy sample may not represent Indigenous populations as a whole. However, Indigenous adults demonstrated similar results to healthy middle-aged Indigenous adults, suggesting that these results are consistent with other healthy Indigenous populations. As Indigenous populations consist of many distinct nations, with their own cultural history, language and beliefs, a single sample of individuals may not accurately reflect all nations [47]. As participants in this investigation self-reported Indigenous ethnicity and were not required to meet minimum status or blood quantum requirements, these results may not fully represent status Indigenous populations or those of minimum ancestry.

## Conclusions

Indigenous adults were found to have healthy cardiovascular structure and function. Sex differences in LV mass and dimensions, and arterial elastance were identified among Indigenous adults. Cardiovascular structure and function may be similar between age- and sex-matched Indigenous and European-descendant adults.
